# Storage of male *Glossina palpalis gambiensis* pupae at low temperature: effect on emergence, mating and survival

**DOI:** 10.1186/s13071-014-0465-y

**Published:** 2014-10-06

**Authors:** Gratian N Mutika, Idrissa Kabore, Andrew G Parker, Marc JB Vreysen

**Affiliations:** Insect Pest Control Laboratory, Joint FAO/IAEA Division of Nuclear Techniques in Food and Agriculture, International Atomic Energy Agency, A-1400 Vienna, Austria

**Keywords:** Chilling, SIT, Sleeping sickness, Tsetse, Trypanosomosis, Competitiveness

## Abstract

**Background:**

Procurement of sterile tsetse flies (*Glossina palpalis gambiensis*) from Burkina Faso for an eradication programme in Senegal that incorporates the sterile insect technique (SIT) required the development of transport and handling protocols that would allow retaining the female flies in the rearing facility and transport of the male flies as irradiated pupae. The proposed handling scheme included the chilling of the male pupae after the female emergence and transport to Senegal under low temperatures. The effect of exposing male pupae of *G. p. gambiensis* to low temperature immediately prior to emergence was investigated.

**Methods:**

The parameters of interest were emergence rate, insemination potential, survival of adult male, male ability to participate in mating activities and productivity of females mated with these males. Production was assessed in laboratory rearing cages and mating behaviour in field cages. Male flies that emerged after the female emergence flush from pupae stored at 10°C or 12.5°C for 5 or 7 days were used in the investigations with flies that emerged under standard colony conditions as control. Males that were 3, 6 or 9 days old competed for mating opportunities with 3 day old females.

**Results:**

The emergence of males after storage of pupae at low temperature (10°C and 12.5°C) for 3, 5, or 7 days was similar to those kept under standard colony conditions while emergence of flies stored at 15°C started before the storage period was over. Survival of males that emerged from pupae stored at low temperature for varying periods was more than 60% at 30 days post emergence (control more than 75%). The fecundity of females inseminated by males that emerged from pupae stored at low temperature for varying periods ranged from 0.33±0.16 to 0.73±0.04 pupae per female per 10 days (control 0.60±0.16). The older males, irrespective of treatment, out-competed the younger males and 3 day- old males transferred lower amounts of seminal contents to the females.

**Conclusions:**

Storage of male pupae at low temperature for periods up to 7 days at the end of the male pupal period could not be directly associated with impairment of mating activity.

## Background

Tsetse flies are obligatory blood feeders that are widespread in sub-Saharan Africa and consequently present a disease threat and biting nuisance to cattle and humans in their ecological niche [1-3]. Several members of the genus *Glossina* are known to be vectors of the protozoan parasites *Trypanosoma* spp., some of which cause the debilitating disease African animal trypanosomosis (AAT) or nagana in livestock and human African trypanosomosis (HAT) or sleeping sickness in humans. Most wild reservoir hosts of *Trypanosoma* spp do not show any apparent negative clinical effect of the presence of the parasites but domesticated hosts and humans often suffer and may succumb to the effects of the protozoan load [4]. In West Africa *Glossina palpalis gambiensis* is one of the important vectors of trypanosomes [5,6]. For over a century studies have been conducted to better understand vector dynamics and disease epidemiology with the intention of lessening or eliminating the disease burden [7-13]. The treatment of confirmed HAT cases has relied on drugs whose mode of action was not fully understood [14], with little advance since the late nineteenth century when livestock owners administered various concoctions to their animals in the hope of curing AAT [15]. Several action programmes have been implemented to control the vector and parasite in efforts to improve the livelihood of communities [4]. The sterile insect technique (SIT) has been part of the arsenal of techniques employed in area-wide control of *G. p. gambiensis* in parts of West Africa [16,17] and considerable efforts have been directed towards improving the application of the technique [18-22].

Recently the Government of Senegal embarked on a project with the ultimate aim of creating a sustainable *G. p. gambiensis* free area in the Niayes, located north-east of Dakar. The area has a coastal microclimate favourable to holding exotic cattle breeds for milk and meat production, but its ecological conditions are also favoured by the tsetse fly *G. p. gambiensis*. A comprehensive feasibility study indicated that (1) suitable tsetse habitat was extremely fragmented, (2) flies were found in very small pockets but sometimes at high densities, (3) the total infested area was limited to 525 km^2^, and (4) no gene flow occurs between the *G. p. gambiensis* populations of the Niayes and the nearest known population in the west of the main tsetse belt of Senegal, which confirmed the isolated nature of the Niayes population. As a result of this study, the Government of Senegal opted for an area-wide integrated pest management (AW-IPM) approach to create a sustainable *G. p. gambiensis*-free zone in the Niayes. Application of insecticide as pour-on on livestock, impregnated in traps, and in netting fences around pig pens were selected as the most appropriate tactics to reduce the high fly population densities, followed by the release of sterile male flies (the SIT) as the final eradication component.

The Government of Senegal opted to procure the sterile flies from the Centre International de Recherche-Développement sur l′Élevage en zone Subhumide (CIRDES) in Burkina Faso, where a colony of the target species has been maintained for the last 40 years [23] rather than establishing and operating a costly national rearing facility to produce the sterile male flies. This decision required the development of transport and handling protocols to retain the female flies in CIRDES for their colony and transport of the male flies as irradiated pupae. The proposed handling scheme included chilling of the male pupae after the female emergence flush and transport to Senegal under low temperatures.

The current report focuses on the assessment of survival and mating ability of males that were exposed to chilling in the late pupal stage (pharate adult) for a period of time necessary to meet the requirements for the adult release protocols described above. It is a contribution to the understanding of the fundamentals of the SIT component of area-wide integrated pest management.

## Methods

### Fly strains

All experiments were carried out with a strain of *G. p. gambiensis* from pupae received from CIRDES in Burkina Faso that was derived from field pupae collected and first colonised in Burkina Faso in 1975 and that has been maintained using an *in vitro* feeding system at the Insect Pest Control Laboratory (IPCL), Seibersdorf, Austria [24,25] since 2009. The colony was fed bovine blood (Svaman spol, s.r.o., Myjava, Slovak Republic), frozen at minus 20°C and irradiated with 1000 Gy in a commercial 220 PBq ^60^Co wet storage panoramic shuffle irradiator. Aliquots of the blood were thawed and used as required. The flies were fed three times a week and maintained under a 12 L:12D light regime cycle, pupae were incubated at 24.1 ± 0.1°C 78.8 ± 3.7% R.H. for four weeks and adults emerged under the same temperature and humidity conditions. The term pupa in this paper also includes the pharate adult stage. These conditions will be referred to as standard colony conditions henceforth.

### Storage of male pupae at low temperature

*G. p. gambiensis* pupae were collected between 09:00 h and 10:00 h every day and incubated in open petri dishes in a Weiss Technik incubation chamber at 24.1 ± 0.1°C and relative humidity 78.8 ± 3.7%. Emergence cages were placed on Petri dishes 28 days post larviposition and emergence of female adults generally started 32–33 days post larviposition. The majority of females emerged within three days after onset of emergence and on day 35 post larviposition the remaining pupae were considered to be male. The male pupae were collected and stored at 10°C, 12.5°C or 15°C in a Luwa ES2000CDM environmental chamber for 3, 5 or 7 days. After the storage period the pupae were placed back in the incubation chamber at 24.1 ± 0.1°C, 78.8 ± 3.7% r.h. for emergence. Each combination of low temperature and period in storage was replicated three times and pupae that were maintained under standard colony conditions (no exposure to low temperature) were used as the control. The term pupa in this paper also includes the pharate adult stage.

The number of adults that emerged from each of 3 replicates of 30 pupae for a 3, 5 or 7 day storage period at 10°C or 12.5°C was recorded daily and emergence rate calculated as the percentage of emerged adults from the total pupae. Only flies that completely pulled away from the puparial case were considered as emerged.

### Survival of males

The adult males that emerged from the different batches were collected into fly holding cages (diameter 20 cm, height 5 cm) with three replicates being observed for each combination of the selected low temperature and period in storage. The flies were fed and maintained under standard colony conditions. Mortality was recorded daily until 30 days post emergence.

### Mating ability of males

The ability of males to engage in mating activity with normal colony females was assessed in fly holding cages in the laboratory. Male pupae were collected and stored at 10°C or 12.5°C for 3, 5 or 7 days. After the storage period the pupae were placed in 24.1 ± 0.1°C, 78.8 ± 3.7% R.H. for emergence. At emergence 15 males and 60 females were placed in colony production cages (diameter 20 cm, height 5 cm) and maintained under standard colony conditions for 30 days. Three replicates for each combination of low temperature and period in storage were run with pupae that were maintained under standard colony conditions (no exposure to low temperature) used as the control. The production of pupae was recorded and female fecundity expressed as the number of pupae per female per ten days (p/f/10d).

### The field cage and its environment

Mating competitiveness of the male flies was assessed using field cages that provide a more natural environment than laboratory cages. The netting cage [26] was cylindrical, 2.9 m in diameter and 2.0 m high [27,28]. The field cage was erected inside a greenhouse under natural light without environmental controls for the age group experiments. When external environmental conditions were cool, the cage was set up under fluorescent lights in a room with ceramic tiles on the floor and walls where temperature was regulated between 24-25°C and humidity 60-65%. Light intensity varied from 290–550 Lux depending on the position in the cage. To monitor environmental conditions, temperature and humidity were recorded every thirty minutes starting at 09:00 h and ending at 12:00 h.

### Effect of age on mating performance

To provide a control for the subsequent experiments, the mating competitiveness of untreated *G. p. gambiensis* males of three different ages was assessed. Virgin males at 3-, 6- and 9-days after adult emergence were released together with 3-day old virgin females in a walk-in field-cage. Forty males of each age group were competing for mating opportunities with 40 females at an initial 3:1 male:female ratio. Flies received a blood meal the day before field cage observation and marking of groups was also done a day before field cage observation. Flies were immobilised in a gentle air flow at 4°C during marking. A small dot of water-based paint was placed on the notum. The unmarked group was also subjected to the low temperature experienced during marking. The experiment was replicated ten times with each replicate lasting from 0900 h to 1200 h.

### Effect of holding male pupae at low temperature on mating performance

Field cage tests were carried out to assess the impact of delaying male adult *G. p. gambiensis* emergence by chilling to simulate the procedure needed to transport male pupae from a rearing facility in one country to a field release programme in another country (in the case of the Senegal project from Bobo Dioulasso, Burkina Faso to Dakar, Senegal). The assumption was that 3–5 days will be required for handling and transportation of the male pupae. Males that emerged under standard colony conditions were used as the control group. Competitiveness was assessed between standard colony males and males from the low temperature treatments (10°C or 12.5°C) of the same post emergence age, i.e. 3-day old standard colony vs 3-day old after low temperature storage, 6-day standard colony vs 6-day old after low temperature storage, and 9-day standard colony vs 9-day after low temperature storage. The emergence of the stored males was delayed by the low temperature so they were most likely physiologically a little older than the control males. Males that were 3 days old were only used for the treatment where pupae were stored at low temperature for seven days and not the 3 and 5 days storage. In all the competition experiments females were 3-days old and emerged under standard colony conditions. Dissections of females were carried out in saline solution and spermathecal contents assessed subjectively at x50 magnification using a Carl Zeiss stereo microscope. The spermathecal value was obtained by assessing the content of the two spermathecae and adding the values. Each spermatheca was classed as empty, 0; quarter full, 0.25; half full, 0.5; three quarters full, 0.75 and full, 1 [29].

### Mating indices and data analysis

Mating indices (propensity of mating, relative mating index and relative mating performance) were used as defined in Mutika *et al*. [28]. The mating propensity (PM) was defined as the overall proportion of released females that mated. It represents the overall mating activity of the flies under the given environmental conditions and is used to assess suitability of the conditions and the flies for the test. The relative mating index (RMI) is a measure of mating competitiveness and was defined as the number of pairs of one treatment as a proportion of the total number of matings; values can range from 0 to 1. In case of equal competition between treatments, all treatments would have equal RMI values adding up to 1. The relative mating performance (RMP) is a measure of mating compatibility and was defined as the difference between the numbers of matings of the control treatment and the other treatment as a proportion of the total number of matings. Values can range from −1 to +1, where positive values indicate preferential mating by the control treatment and negative values preferential mating of the pupae stored at low temperature and/irradiated treatments. Data for premating time, mating duration and spermathecal value were tested for normality and homoscedasticity (Anderson-Darling test and Levene’s test in Minitab) followed by either analysis of variance or Kruskal-Wallis analysis including multiple comparisons. Data was pooled for Kruskal-Wallis analysis. The Bonferroni Z-value was used in the pairwise comparisons with a family error rate of 0.2 leading to a desired confidence of 96.7%. A two-way analysis of variance followed by pairwise comparisons was carried out on the number of mating pairs in the field cage [30]. Binary logistic regression analyses were carried out on emergence rate and survival of *G. p. gambiensis* after exposure to low temperature for varying periods. A regression analysis was carried out for the fecundity data. Normal probability plots of the residuals were used to assess normality of distribution [31,32].

## Results

### Storage of male pupae at low temperature

**Figure 1:**
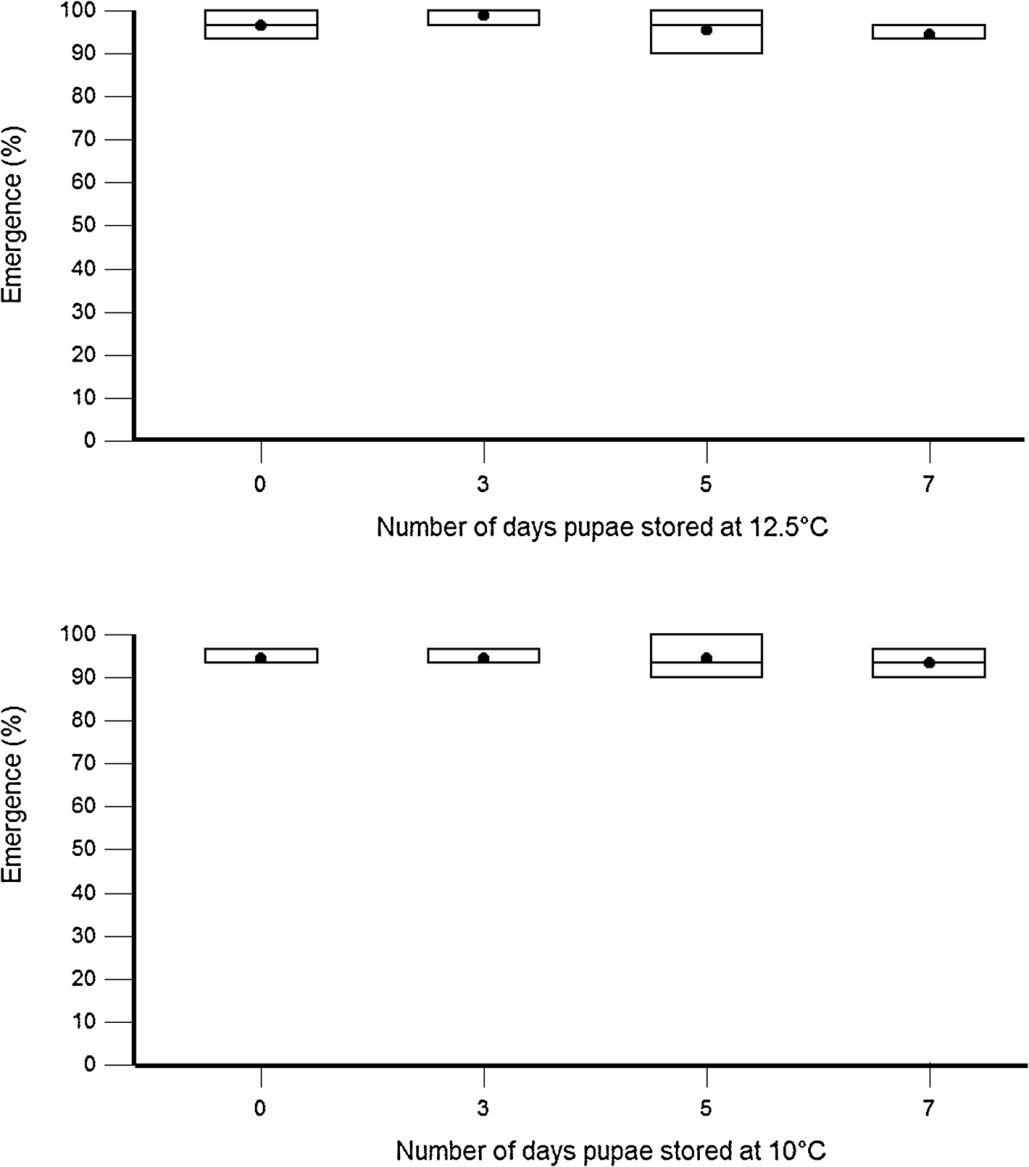
**The emergence of** ***Glossina palpalis gambiensis*** **after storing the pharate adults at 10°C or 12.5°C for 3, 5 or 7 days compared to standard colony conditions.** The top and bottom of the boxplot are 95% CI, line across the box is median, dot in the box is mean and whiskers show the lowest and highest data points.

The mean emergence rate was above 90% for all treatments and was not significantly affected by the cold storage (Z = −1.21; P > 0.05; odds ratio 0.86). The temperature at which the pupae were stored did not have a significant effect on emergence rate (Z = 1.21; P > 0.05; odds ratio 1.22) (Figure 1).

Adult flies continued to emerge when pupae were stored at 15°C with over half the adults in the samples emerging by the 7^th^ day in storage. Tests at 15°C were thus discontinued.

**Figure 2:**
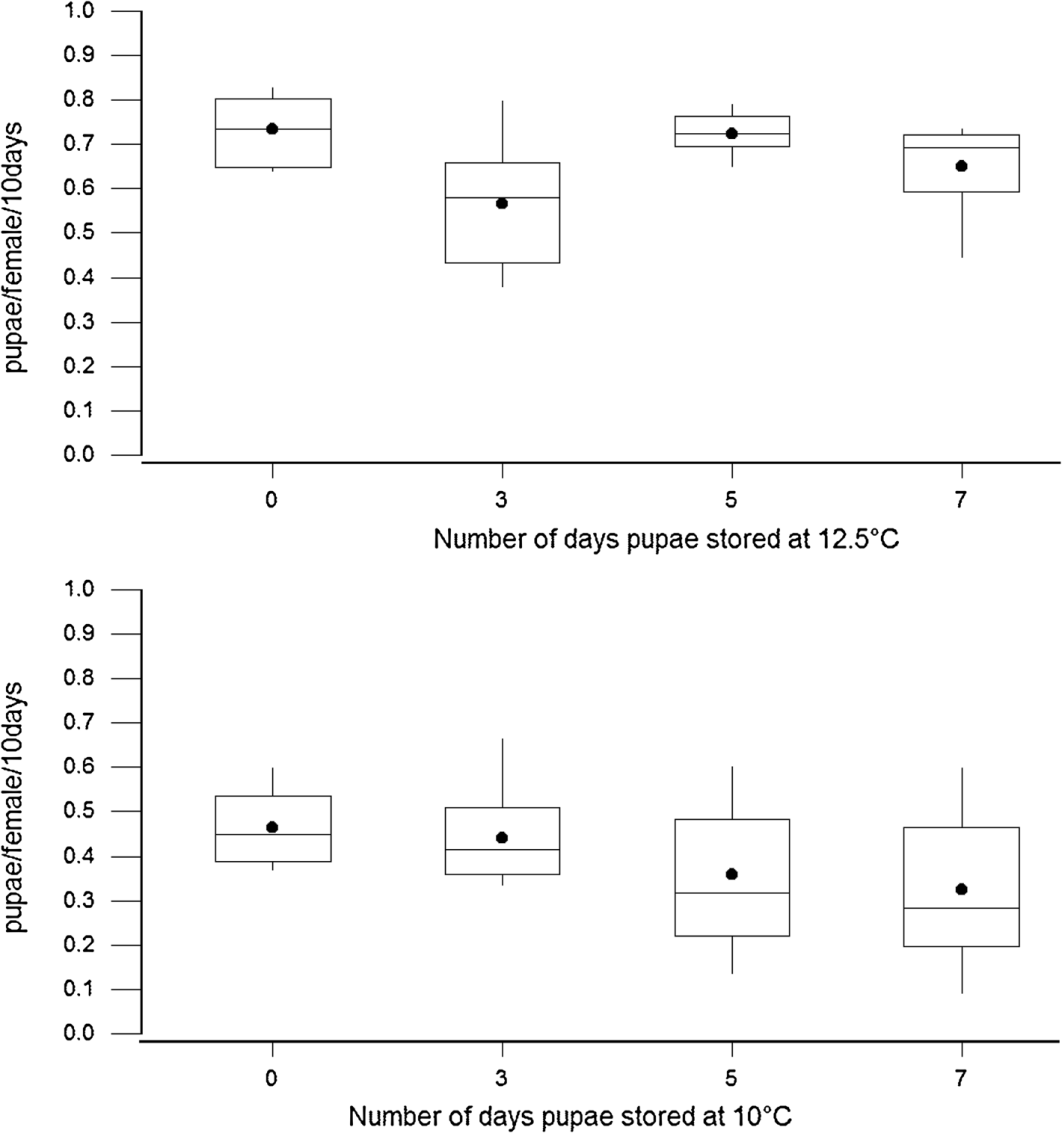
**The survival at 30 days post emergence of male** ***G. p. gambiensis*** **stored at 10°C or 12.5°C for 3, 5 or 7 days.** The top and bottom of the boxplot are 95% CI, line across the box is median, dot in the box is mean and whiskers show the lowest and highest data points.

There was a significant deviance from zero for at least one survival predictor (G = 54.854, df = 3 and P < 0.001) at storage temperature of 10°C or 12.5°C for 3 days storage. The storage of pupae at 10°C for 3 days significantly lowered the survival of males 30 days after emergence (Z = −3.48; P < 0.001; odds ratio 0.42). Survival of males after exposure to low temperature as pupae was not significantly affected by the length of storage (Z = 1.11; P > 0. 05; odds ratio 1.07) (Figure 2) but survival rates were significantly lower when the flies were older than soon after emergence (Z = −5.80; P < 0.001; odds ratio 0.94) and lower temperature (Z = −2.47; P < 0.05; odds ratio 0.41).

**Figure 3:**
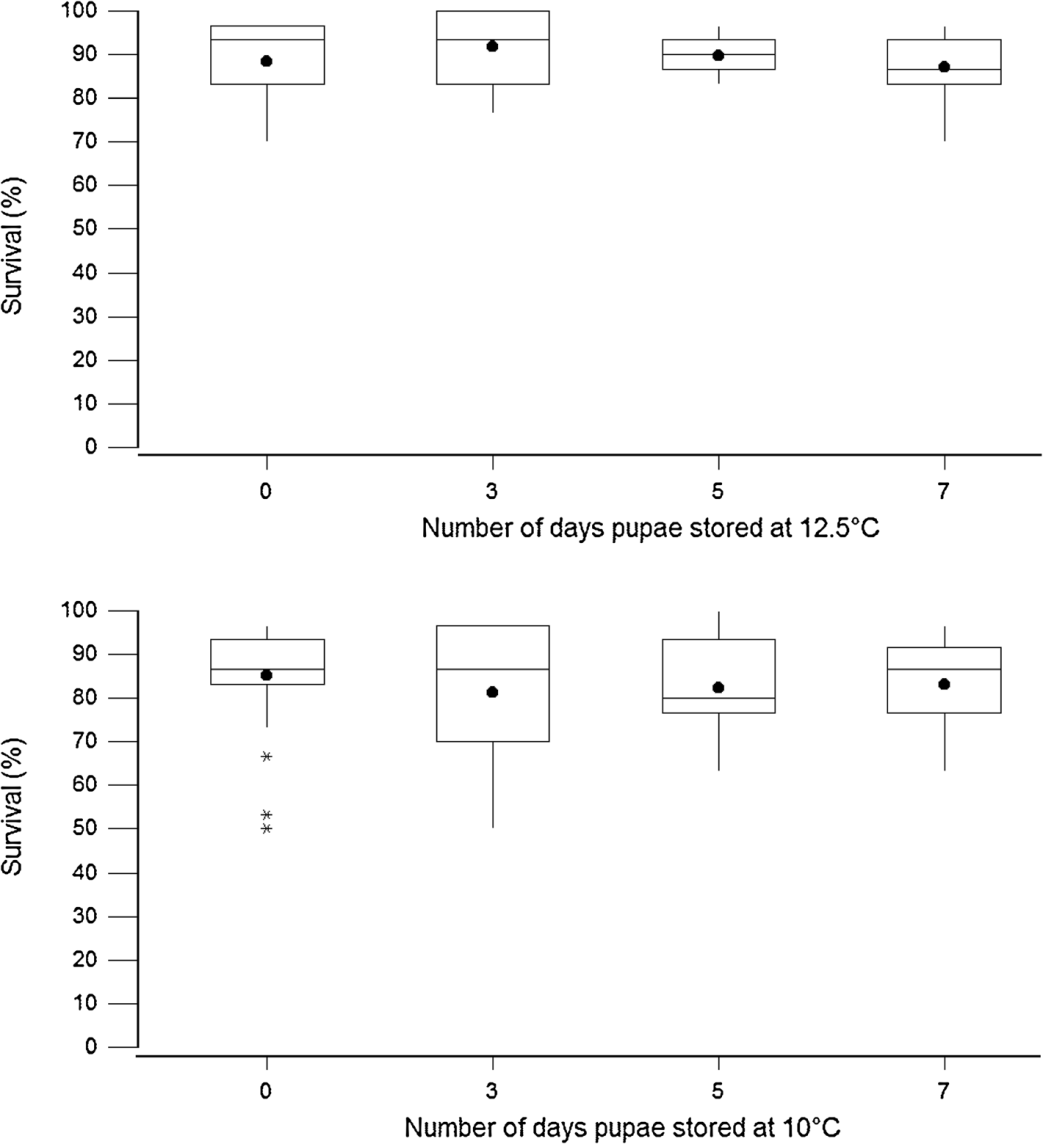
**The fecundity, pupae per female per 10 days** ***G. p. gambiensis*** **inseminated by males stored at 10°C or 12.5°C at the end of pupal stage and adults maintained in standard colony conditions.** The top and bottom of the boxplot are 95% CI, line across the box is median, dot in the box is mean and whiskers show the lowest and highest data points

Pupae production of females mated with males that emerged from pupae stored at low temperature was slightly lower than with normal colony males, although not significantly affected by storage at 10°C or 12.5°C for up to 7 days (Figure 3). There was a poor fit of the regression model, R-Sq (adj) = 10% with a significant regression (F = 4.95; df = 2,69; P < 0.01) and the linear predictors contribute very little to the variation in pupae production: pupae per female per 10 days = 0.222 + (0.017*days in storage) + (0.0172*temperature in storage).

### Effect of age on mating performance of untreated males in field cages

Temperature in the greenhouse ranged from 18°C at the start to 36°C at the end of observations, relative humidity ranged from 67% down to 26%. There was hardly any flight activity below 20°C with the exception of flight immediately after release. The pre-mating time appeared to be dependent on temperature in the green house with most mating activity taking place when temperature was above 24°C for the three age groups. Most flies rested in darker areas on the lower portions of the cage or in shady places, for example under the seat used by the observer when the temperature rose above 27°C. Mean mating propensity (±se) was 0.83 ± 0.06 with 0.4 being the least recorded for all the replicates. The 9- and 6-day old males were significantly (F = 38.46, df = 2,18, P < 0.01) more competitive than 3-day old males, with the greatest number of mating pairs involving the oldest males and the youngest having the least number of pairs. The Relative Mating Index (±se) was 0.13 ± 0.02 (3 days); 0.36 ± 0.02 (6 days) and 0.51 ± 0.02 (9 days). If the competitiveness of the males of the three ages tested was the same then 0.33 of the mating pairs would be from each age. The premating time was similar for the three age groups (F = 0.44; df = 2,247; P > 0.05), mating duration was significantly lower for 3 day old males than in 9 day old males (F = 4.03; df = 2,246; P < 0.05) and spermathecal fill was significantly lower for 3 day old males than in 6 and 9 day old males (H = 24.47; df = 2; P < 0.001) (Table 1).

**Table 1:** **Pre-mating period and duration of mating in minutes (mean ± se) of unchilled** ***Glossina palpalis gambiensis*** **males of different ages and spermathecal value (mean ± se) for the females that mated with the males**

**Male age**	**N**	**Premating period** ^**1**^	**Mating duration** ^**1**^	**Spermathecal value** ^**1**^
3 days	33	102.2 ± 11.2^a^	59.5 ± 4.27^a^	1.19 ± 0.15^a^
6 days	88	102.5 ± 6.25^a^	68.6 ± 2.64^b^	1.76 ± 0.06^b^
9 days	129	95.3 ± 5.36^a^	73.2 ± 2.22^b^	1.78 ± 0.05^b^

^1^Values followed by the same letter in the same column are not significantly different at α = 0.05.

### Effect of holding male pupae at low temperature on mating performance

#### 10°C for five days

The proportion of 6- and 9-day old males that mated when pupae were stored at low temperature for 5 days or not exposed to low temperature were similar (F = 2.63; df = 3, 27; P > 0.05). Premating time was significantly shorter for 9 day old controls and chilled males compared to 6 day old males. The median mating duration was shorter for 6 day old males for both treatments and longer for 9 day old males. Spermathecal fill was similar for both ages, control and chilled at nearly full (Table 2).

**Table 2:** **Median (96.7% CI) pre-mating period and duration of mating in minutes of** ***Glossina palpalis gambiensis*** **males emerged from chilled pupae and age-matched adults from unchilled pupae and spermathecal value for the mated females**

**Male pupae treatment**						
**Age (days)**	**Temperature (°C)**	**Storage (days)**	**Mating pairs**	**Premating period**	**Mating duration**	**Spermathecal value**
3	24	0	100	51.5 (35,71)	70.5 (67,80)	2
6	24	0	393	17 (14,22)	77 (74,80)	2
9	24	0	457	6 (5,7)	79 (77,83)	2
6	10	5	121	11 (4,14)	76 (72,81)	2
9	10	5	174	4 (3,5)	81.5 (76,88)	2
3	10	7	33	31 (11,62)	82 (64,92)	2
6	10	7	48	28 (13,58)	76 (67,87)	2
9	10	7	84	14 (10,28)	85.5 (80,92)	2
6	12.5	5	44	24 (15,43)	76.5 (72,85)	2
9	12.5	5	72	6 (4,8)	76 (73,81)	2
3	12.5	7	28	59.5 (16,74)	67 (57,72)	2
6	12.5	7	56	28.5 (19,43)	81.5 (75,88)	2
9	12.5	7	75	10 (7,15)	75 (70,78)	2

#### 10°C for seven days

The 9 day old control and chilled males were equally competitive with fewer mating pairs of 3- and 6- day old chilled males than control males being recorded. There were significantly more mating pairs recorded for older males than the younger males irrespective of whether they were chilled or not (F = 4.80; df = 5,20; P < 0.01). Premating time was significantly shorter for 3 day old chilled males compared to control males of similar age (Table 2). Overall the 9 day old mated significantly earlier than the 3 and 6 day old males. The median mating duration was similar for 3 and 6 day old males for both treatments and significantly longer for 9 day old chilled males. Males of the three ages, control and chilled could inseminate the females (Table 2).

#### 12.5°C for five days

The proportion of 6 and 9 day old males that emerged from pupae stored at low temperature for 5 days that mated was similar to males that emerged from pupae stored under standard colony conditions (F = 1.69; df = 3,12; P > 0.05). Older male flies that were stored for 5 days at 12.5°C mated significantly earlier than younger males stored at the same temperature for the same length of time (Kruskal-Wallis multiple comparisons Z = 3.2718; z-critical value = 3.016; P < 0.01). Copulation duration of the males that emerged from pupae that were stored at low temperature was similar with the 6 and 9 day old control males (Table 2). Spermathecal fill was similar for females mated with 6 and 9 day old males irrespective of whether the pupae were stored at low temperature or not. The median spermathecal values were the same for the females that mated with 6- and 9- day old males.

#### 12.5°C for seven days

There were significantly (F = 7.34; df = 5,20; P < 0.001) more mating pairs recorded for older males than the younger males irrespective of whether they were chilled or not (Table 3). The numbers of mating pairs were highest for 9 day old males, followed by 6 day old males, with fewest pairs recorded for 3 day old males. Within each age group fewer chilled males secured mating opportunities than standard colony males although not significantly different. The median premating time was significantly shorter for chilled 3 day old males (Kruskal-Wallis multiple comparisons Z = 3.9764; z-critical value = 3.016; P < 0.001) compared to 9-day old males and similarly for 6- (Kruskal-Wallis multiple comparisons Z = 3.4936; z-critical value = 3.016; P < 0.01) compared to 9- day old chilled males. The median mating duration was similar for 6- and 9- day old males for both treatments with the 3 day old chilled males mating for a shorter duration (Kruskal-Wallis multiple comparisons Z = 3.12387; z-critical value = 3.016; P < 0.01) than the 6-day old males and 9 –day old standard colony males (Kruskal-Wallis multiple comparisons Z = 3.3161; z-critical value = 3.016; P < 0.01). Mating duration was nearly the same for control and chilled flies and when insemination occurred it was frequently complete filling of spermathecae for both treatments.

**Table 3:** **The relative mating indices for** ***Glossina palpalis gambiensis*** **in a field cage for different ages following chilling of male pupae**

**Male age and pupal treatment**				**Mating propensity**	**Relative mating index (±se)**
**Age (days)**	**Temperature (°C** **)**	**Storage (days)**	**n**		**Chilled**
6	10	5	10	0.705 ± 0.05	0.432 ± 0.07
9	10	5	10	0.925 ± 0.02	0.471 ± 0.04
3	10	7	5	0.415 ± 0.04	0.397 ± 0.05
6	10	7	5	0.650 ± 0.07	0.362 ± 0.02
9	10	7	5	0.840 ± 0.05	0.510 ± 0.08
6	12.5	5	5	0.600 ± 0.13	0.341 ± 0.05
9	12.5	5	5	0.775 ± 0.08	0.464 ± 0.08
3	12.5	7	5	0.39 ± 0.07	0.322 ± 0.08
6	12.5	7	5	0.650 ± 0.07	0.457 ± 0.08
9	12.5	7	5	0.845 ± 0.05	0.444 ± 0.02

## Discussion and conclusion

Physiology is an important factor in the application of the sterile insect technique for tsetse flies. This is important since there may be a need for manipulation of insects in conditions not normally experienced in nature. The manipulation may include storage of some life stages at low temperature and may in the worst case scenario lead to severe negative effects on their biological quality. Some insects enter a diapause phase during harsh seasons or pass the hostile seasons in life stages that are more resilient to adverse conditions but this is not observed in tsetse [33]. Generally when insects that spend a large proportion of their lives in warm environments are exposed to low temperature all physiological processes may be slowed down. For insects that do not diapause this may be accompanied by irreparable damage if exposure is prolonged.

Normal development in tsetse flies is thermodynamically controlled and prolonged exposure to extreme temperatures for any of the life stages is potentially lethal [34-36]. However, brief exposure to low temperature may be included during processing e.g. to separate the sexes, thus sacrificing some of the insects’ quality in order to bring about the desired process efficiency [37-39]. In the application of the SIT, the quality of the males is of prime importance since the males will be expected to successfully compete against wild males for mating opportunities [40].

Field observations have shown that there must be a very efficient mating system in most *Glossina* spp*.* as the majority of females are inseminated within a few days after emergence [41-44], although aside for the *G. morsitans* group, there are few records of where mating takes place. The application of the SIT directed at suppression or eradication of wild tsetse populations would thus need logistical arrangements that minimize changes in mating behaviour. In mass-rearing facilities male and female flies are placed in the same cages after emergence and therefore the males do not have to actively seek females to mate [24,45-47]. In nature the situation is different and the released males have to locate females willing to mate whilst competing for mating opportunities with wild males. In the case of *G. austeni* on the Island of Unguja, data is available that indicates that the distribution of the released sterile males was highly correlated with that of wild male and female flies. The ability of sterile males to aggregate (and thus locate) in those areas preferred by the wild males is of primary importance to ensure adequate sterile-to-wild male ratios everywhere [40].

Based upon our observations, it is important to consider the balance between increasing competitiveness with age and age specific mortality, as well as the cost of keeping males in the insectary for a longer period, in order to enhance chances of delivering sterile sperm to the wild population. The higher occurrences of mating pairings with older males could indirectly be taken to indicate that older males engaged in flight activity more frequently than the younger males although there was no systematic measurement of flight events. Laboratory investigators using small rearing cages have noted that lower proportions of young males of *Glossina* spp. win mating opportunities when in direct competition with older males [29,48-52]. The mating competition results are in line with work carried out with *G. f. fuscipes* and *G. p. palpalis* in field cage observations that showed that young males can win mating opportunities when competing against older males albeit in a lower ratio compared to older males [53]. In the application of SIT, ideally the released males would be equally competitive with the wild males for the technique to have any impact on the wild population. With the release of a higher ratio of sterile: wild males, ensuring a numerical advantage for the sterile males, it would be expected that more sterile matings than wild matings occur leading to a reduction in the wild population [54].

The viability of sperm of males that emerged from pupae that had been exposed to low temperature was maintained as supported by the female productivity data. The slightly lower productivity can be attributed to other factors other than young age since field cage observations clearly demonstrated adequate transfer of seminal contents irrespective of pupal treatment. Contrary to some observations [55] exposure of the pupae to low temperatures of 10°C and 12.5°C for up to seven days allowed for accelerated emergence upon return to standard colony conditions with negligible effects on the mating competitiveness of the adult flies. The survival and willingness of adults to feed prior to the field cage tests was an indication of the level of similarity to pupae handled in standard colony conditions. Low levels of mating activity were recorded at low temperature and low light intensity which may loosely correspond to some diurnal preferences noted in the general seasonal behaviour of *G. p. gambiensis* [56].

The data of this paper indicate that transport of male *G. p. gambiensis* pupae that are maintained at 10°C and 12.5°C enables the possibility of shipping tsetse pupae between distant points within a country and across national boundaries while being able to reserve a large proportion of female flies for colony production. The possibility of storing pupae for several days enables project managers to either accumulate reasonable quantities of adult tsetse flies for dispersal or simply to be flexible in scheduling dispersal.

In this paper the biological quality of males that were exposed to low temperature is confirmed by the successful extrication from the puparium indicated by the emergence rate above 90%, survival rate above 60% at the end of four weeks and ability to inseminate colony females resulting in pupae production above 0.3 p/f/10 days, which is a sufficient rate to at least maintain colony size. In addition the males that were stored at low temperature at the end of the pupal stage were also shown to exhibit acceptable mating activity in the field cage with premating time, mating duration and ability to inseminate females, comparing favourably with control males. Based upon the data presented in this paper, a handling and shipping protocol was developed to supply the Senegal tsetse eradication project with male pupae from Burkina Faso. During weekly shipments, the project received male pupae that were chilled at 10°C and transported in insulated transport boxes that maintained the temperature around 10°C using phase change packs for several days.

The performance of the released male flies in the field is, however, dependent on many other factors. The results of the trial releases in the target area in Senegal with male flies that were transported as pupae using the described protocol are the subject of another publication. The laboratory results presented in this paper are in line with a recent field assessment using the same *G. p. gambiensis* strain that confirmed that the strain can still compete favourably against wild males after being mass-reared for over four decades [23].

Storage of male pupae at low temperature for periods up to 7 days at the end of the male pupal period could not be directly associated with impairment of mating activity. These experimental results support the idea that the chilling of flies at the late pupal stage can be used as a way of improving logistics during an SIT for tsetse programme.
